# Generation and Characterization of the Human Anti-HTNV Antibody KJJ4

**DOI:** 10.3390/ijms27187994

**Published:** 2026-09-08

**Authors:** Ziyan Chen, Yanbo Wang, Yongli Hou, Liang Fang, Linfeng Cheng, Yusi Zhang, Chunmei Zhang, Yun Zhang, Ying Ma, Kang Tang, Lihua Chen

**Affiliations:** 1 Department of Immunology, The Fourth Military Medical University, Xi’an 710032, China; zyanchen@fmmu.edu.cn (Z.C.); hzcm1981@126.com (C.Z.);; 2 Department of Microbiology, The Fourth Military Medical University, Xi’an 710032, China

**Keywords:** Hantaan virus (HTNV), neutralizing antibodies (NAbs), IgG4, glycoprotein Gn, in vitro neutralization

## Abstract

Hantaan virus (HTNV) is the predominant causative agent of hemorrhagic fever with renal syndrome (HFRS) in China, yet no specific antiviral therapy is currently available. Neutralizing antibodies (NAbs) represent a promising strategy, but most existing anti-HTNV NAbs are heterologous and carry immunogenicity risks. Here, two fully human antibodies from a previously established human anti-HTNV phage display library were generated and characterized. KJJ3, a VL-VL tandem antibody derived from clone 3–12, showed weak binding to inactivated HTNV and minimal neutralizing activity (IC_50_ = 27.12 μg/mL). However, KJJ4, an engineered IgG4 antibody derived from clone 4–19 and carrying the S108P hinge mutation to prevent Fab-arm exchange, bound inactivated HTNV antigen and recombinant glycoprotein Gn (residues 19–371) in a dose-dependent manner, with only weak binding to Gc. Surface plasmon resonance yielded an association rate constant of 4.70 × 10^3^ M^−1^s^−1^, a dissociation rate constant of 3.06 × 10^−3^ s^−1^, and an equilibrium dissociation constant of 650 nM for monomeric Gn_19–371_. In a Vero E6 focus-reduction microneutralization assay, KJJ4 neutralized HTNV in vitro with an IC_50_ of 2.879 μg/mL. These data establish KJJ4 as a fully human anti-HTNV antibody with experimentally defined in vitro binding and neutralizing activity, warranting further evaluation in animal models of HTNV infection.

## 1. Introduction

Hantavirus (HTV) is a member of the genus Orthohantavirus within the family Hantaviridae (order Bunyavirales) and is an enveloped, single-stranded, negative-sense RNA virus [1,2]. In humans, HTV infection can lead to two distinct clinical syndromes: hemorrhagic fever with renal syndrome (HFRS) and hantavirus cardiopulmonary syndrome (HCPS), with a case fatality rate of up to 15%; consequently, it poses a persistent and significant public health threat globally [3,4]. Notably, the predominant HTV strain circulating in China is the prototype Hantaan virus (HTNV), which causes severe HFRS in infected individuals [5,6,7], and China bears the heaviest global burden of HFRS, consistently accounting for more than 90% of all reported cases worldwide [5,8,9]. HTNV is primarily transmitted to humans via inhalation of aerosolized excreta from infected rodents, rather than by direct person-to-person spread [2,3,4]. Statistical models built on high-resolution climate and population data show that the risk of HFRS in China will persist at a relatively high level by the end of this century [10]. However, the underlying pathogenesis of HFRS remains incompletely elucidated, which severely hinders the development of effective prevention and treatment strategies for this disease. Although inactivated vaccines against HTNV/HFRS have been deployed in China, their protective efficacy and durability remain limited, and no broadly approved specific antiviral therapy is available [1,11,12]. Clinical management relies primarily on supportive care, which has limited therapeutic efficacy but also imposes substantial demands on medical resources [11,13]. Against this backdrop, human-derived anti-HTNV neutralizing antibodies (NAbs), which can block viral entry, have emerged as a potential therapeutic approach [14,15,16,17,18].

The HTNV genome comprises three segments, designated S, M, and L, which encode the nucleocapsid protein (NP), the glycoprotein (GP) precursor (which matures into Gn and Gc), and the RNA-dependent RNA polymerase (RdRp), respectively [19,20]. Among these, the structural proteins NP and GP of HTNV exhibit strong immunogenicity and can induce both cellular and humoral immune responses in the host [21,22,23]. NAbs can specifically bind to Gn, Gc or block HTNV infection of cells [21,24,25]. Neutralizing antibodies against the surface glycoproteins, particularly Gn, are associated with protection in animal models and with recovery from HFRS. However, most of the existing anti-HTNV antibodies with high neutralizing activity are of heterologous origin, which may trigger an immune response against these NAbs in the host, reducing their therapeutic efficacy [24,26]. In contrast, human-derived NAbs have extremely low immunogenicity, can avoid eliciting an immune response against them, and offer high safety in clinical use [14,15,16]. Therefore, screening and generating human-derived anti-HTNV neutralizing antibodies may provide a therapeutic option for patients with HTNV-associated HFRS.

In our previous study, we constructed a human anti-HTNV phage antibody library and preliminarily screened anti-HTNV-specific Fab fragments with neutralizing activity [27]. Building on this foundation, the present study aimed to express these antibodies in a eukaryotic system, evaluate their binding to HTNV antigens and recombinant Gn, and assess their neutralizing activity in a Vero E6 cell-based microneutralization assay. To this end, we analyzed and engineered the Fab fragment gene sequence to generate the V_L_-V_L_ antibody KJJ3 (derived from clone 3–12) and the IgG4 (S108P) antibody KJJ4 (derived from clone 4–19), using the HEK293 eukaryotic expression system. KJJ3 exhibited weak binding to inactivated HTNV and minimal neutralizing activity (IC_50_ = 27.12 μg/mL, approximately 9-fold weaker than KJJ4); KJJ4 was shown to bind HTNV-Gn and neutralize HTNV in vitro, with an equilibrium dissociation constant (*K_D_*) of 650 nM for Gn and an IC_50_ of 2.879 μg/mL. Collectively, these in vitro binding and neutralization data support further investigation of KJJ4 as a candidate antibody against HTNV.

## 2. Results

### 2.1. Generation and Biochemical Characterization of KJJ3 and KJJ4 Antibodies

In previous studies, phage display technology was used to preliminarily screen human anti-HTNV Fab fragments “3–12” and “4–19” with neutralizing activity [27]. Subsequently, DNA was extracted from the corresponding phages and sequenced to obtain the variable region gene sequences of the V_L_ and V_H_ of the Fab 3–12 and Fab 4–19. Because clone 3–12 contained a stop codon in the VH CDR, a VL-VL comparator antibody (KJJ3) was constructed via a flexible (G_4_S)_3_ linker (GGGGS-GGGGS-GGGGS) (Supplementary Tables S1 and S2). The KJJ4 antibody was designed based on the gene sequence of Fab 4–19, with the S108P mutation introduced to eliminate Fab-arm exchange [28,29,30]. Light chain and heavy chain expression vectors were constructed separately (Supplementary Table S3 and S4).

**Figure 1:**
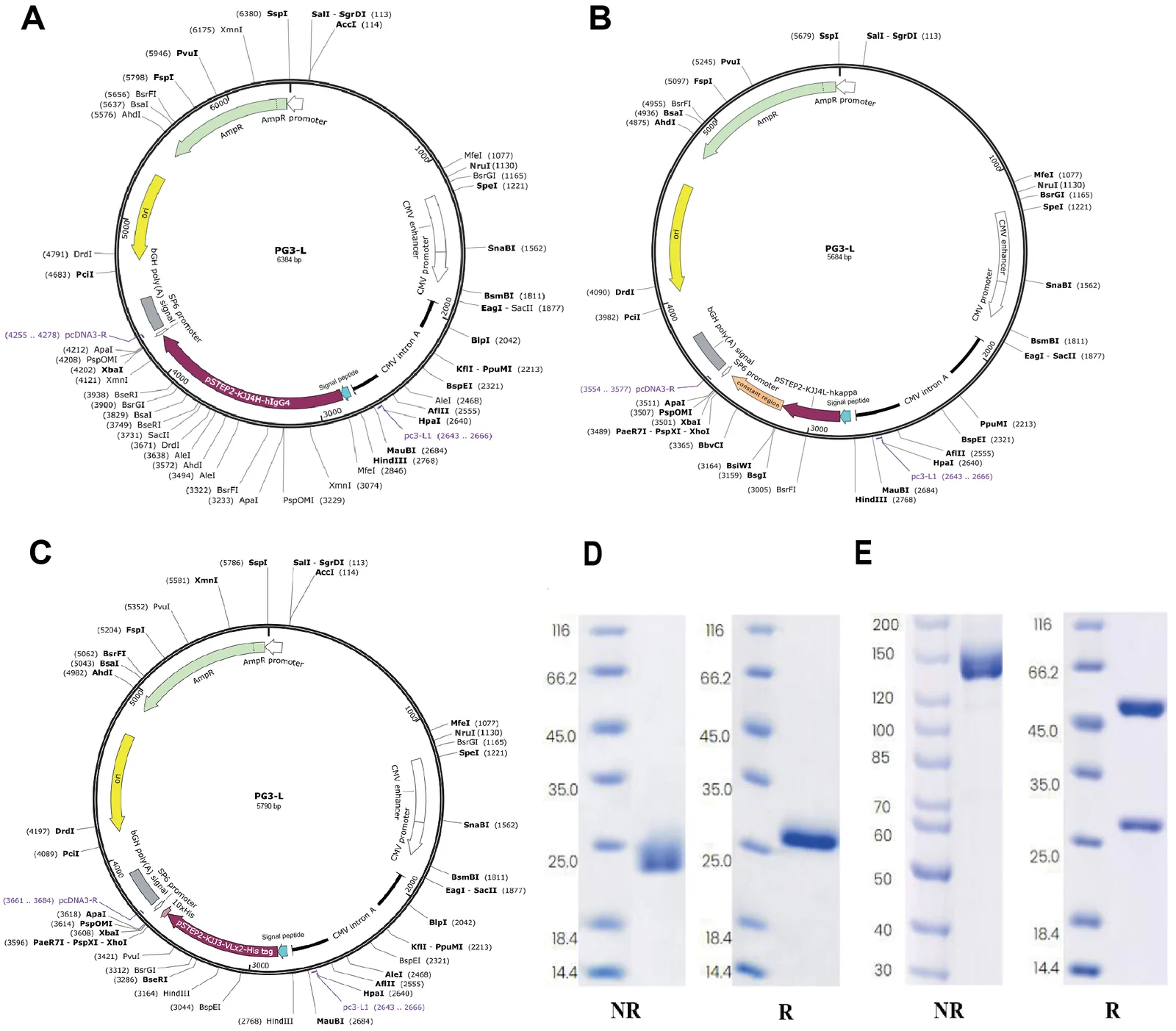
Construction, expression, and purification of recombinant antibodies KJJ3 and KJJ4. (**A**–**C**) Maps of recombinant expression plasmids. (**A**) pPG3-L-KJJ3 encoding the V_L_-V_L_ single-domain antibody KJJ3 with a C-terminal 10 × His tag. (**B**) pPG3-L-KJJ4-LC encoding the KJJ4 light chain. (**C**) pPG3-L-KJJ4-HC encoding the KJJ4 heavy chain of the IgG4 subtype carrying the S108P mutation. (**D**) SDS-PAGE analysis of purified KJJ3 under non-reducing (NR) and reducing (R) conditions. The leftmost lane in each panel shows the molecular weight marker. (**E**) SDS-PAGE analysis of purified KJJ4 under non-reducing and reducing conditions. The leftmost lane in each panel shows the molecular weight marker.

The target genes were amplified by PCR, cloned into the corresponding expression vectors, and the constructs were verified by sequencing. After sequence confirmation, the plasmids were amplified for subsequent experiments. The maps of the recombinant expression plasmids pPG3-L-KJJ3, pPG3-L-KJJ4-LC, and pPG3-L-KJJ4-HC are shown in Figure 1A–C. Following transfection of HEK293 cells, serum-free culture, and affinity purification, the target antibodies were obtained. Elution peak samples were analyzed by SDS-PAGE to assess purification efficiency. KJJ3 migrated as an approximately 25 kDa band under both non-reducing and reducing conditions (Figure 1D). As shown in Figure 1E, under non-reducing conditions, KJJ4 displayed a band at approximately 150 kDa corresponding to intact IgG4; under reducing conditions, it dissociated into two specific bands at approximately 25 kDa (light chain) and 50 kDa (heavy chain), confirming correct antibody assembly. ImageJ analysis of Coomassie Blue-stained bands indicated purification purities of 94.8% for KJJ3 and 99.8% for KJJ4. BCA assay determined concentrations of 2.3 mg/mL and 1.02 mg/mL, respectively. In summary, plasmids carrying the KJJ3 and KJJ4 gene sequences were constructed, and the corresponding antibodies were expressed using the HEK293 eukaryotic expression system, with subsequent functional analyses focusing primarily on KJJ4.

### 2.2. KJJ4 Binds to HTNV Antigen and Exhibits Neutralizing Activity In Vitro

**Figure 2:**
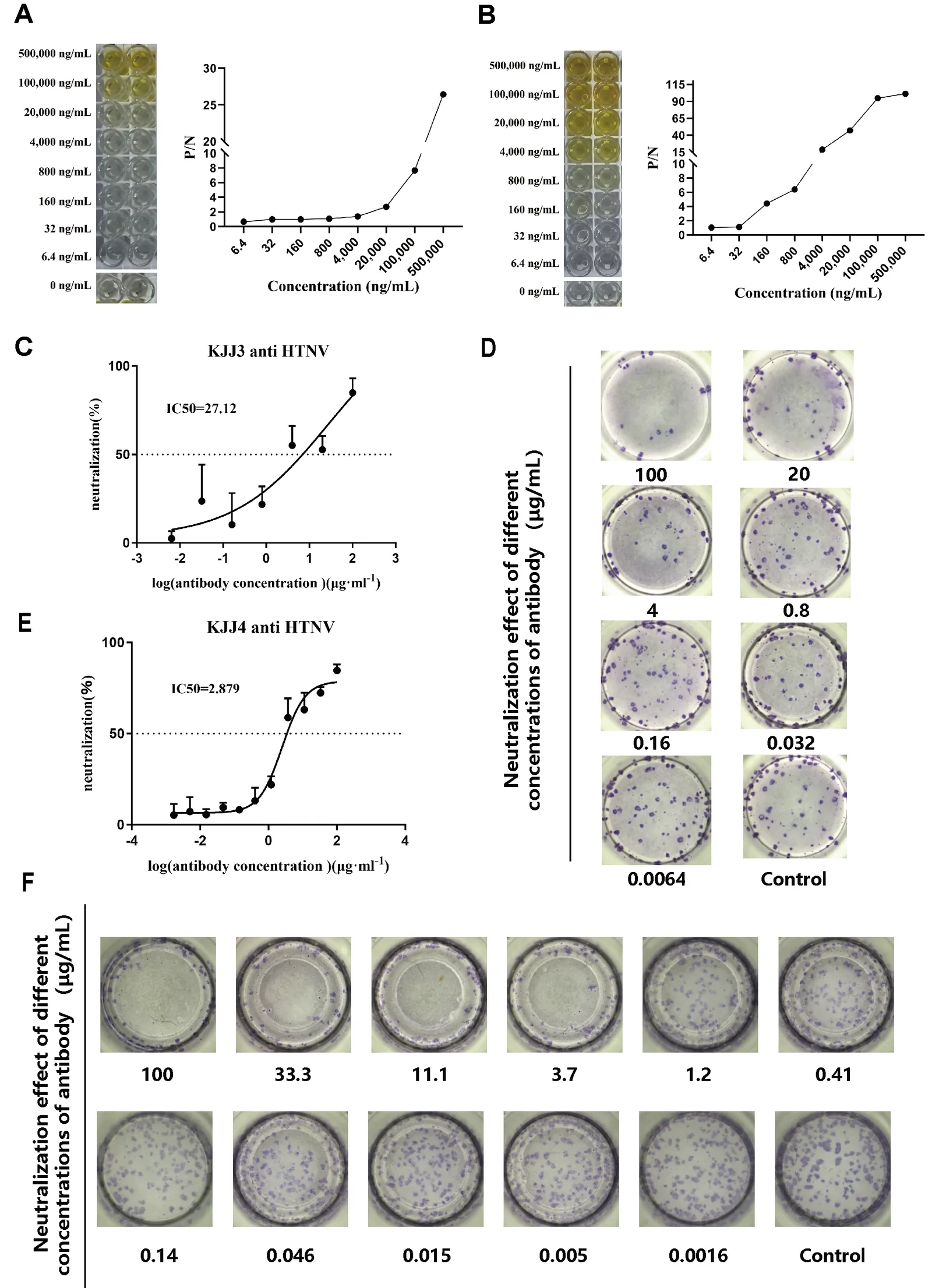
Binding capacity and neutralizing activity of KJJ3 and KJJ4 against HTNV. (**A**) ELISA detection of KJJ3 binding to inactivated HTNV antigen. (**Left**) representative well images at the indicated antibody concentrations; well images are shown for two replicate wells at each concentration. (**Right**) corresponding P/N ratios. (**B**) ELISA detection of KJJ4 binding to inactivated HTNV antigen. (**C**) Dose–response neutralization curve of KJJ3 against HTNV. (**D**) Representative images of viral foci from the microneutralization assay for KJJ3 at the indicated concentrations (μg/mL). (**E**) Dose–response neutralization curve of KJJ4 against HTNV; the IC_50_ was 2.879 μg/mL. (**F**) Representative images of viral foci from the microneutralization assay for KJJ4 at the indicated concentrations (μg/mL). Data are presented as mean ± SEM from three independent experiments (*n* = 3).

After purification, the binding characteristics of the antibodies were evaluated by ELISA using inactivated HTNV antigen. KJJ3 showed only weak binding to inactivated HTNV (Figure 2A). In contrast, KJJ4 exhibited much stronger binding activity: at concentrations ≥ 160 ng/mL, the P/N ratio reached 4.4 (Figure 2B). To rule out the possibility that antigen inactivation might disrupt native conformation and compromise binding results, functional activity was further assessed by a microneutralization assay. KJJ3 displayed only minimal neutralizing activity against HTNV (IC_50_ = 27.12 μg/mL; Figure 2C,D) and was therefore not pursued further. In contrast, KJJ4 inhibited HTNV infection of Vero E6 cells in a dose-dependent manner, with an IC_50_ of 2.879 μg/mL (Figure 2E,F). Thus, KJJ4 possesses neutralizing activity against HTNV in vitro. Given the weak binding and neutralizing activity of KJJ3, subsequent experiments focused on KJJ4.

### 2.3. Specific Binding and Binding Kinetics of KJJ4 for HTNV-Gn

**Figure 3:**
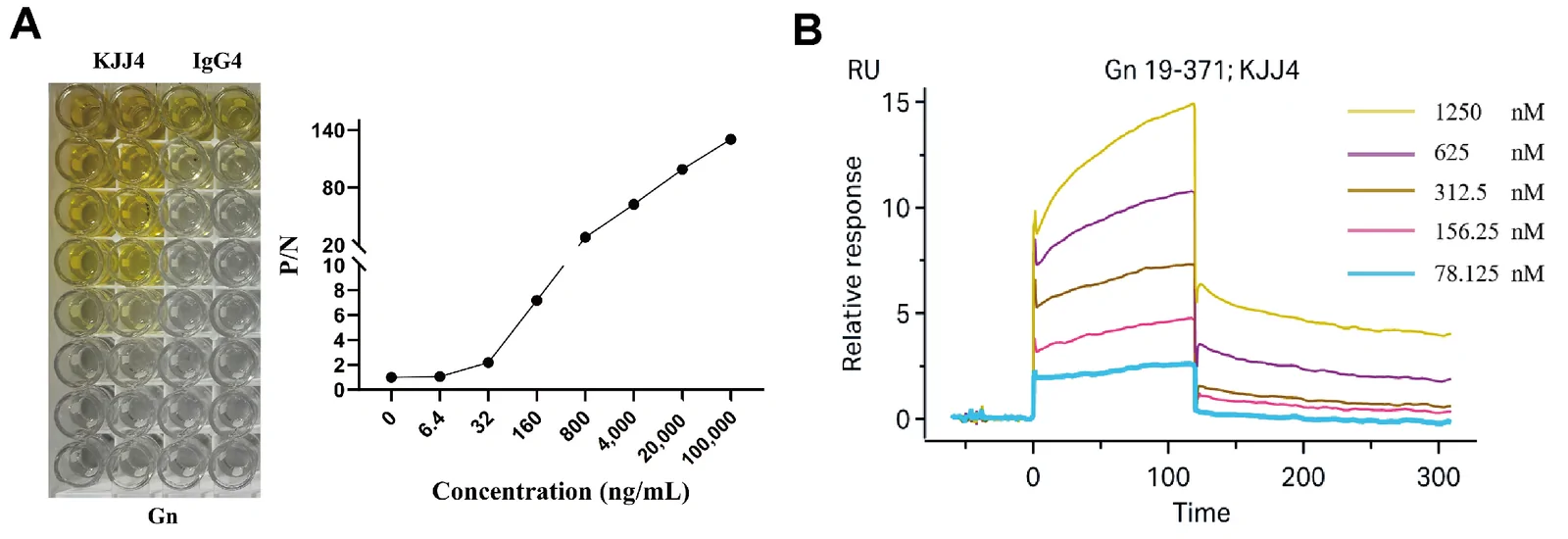
Specific binding of KJJ4 to HTNV-Gn_19–371_. (**A**) ELISA detection of KJJ4 binding to recombinant HTNV-Gn_19–371_. Left: representative wells at the indicated antibody concentrations (ng/mL); the two columns are replicate wells. Right: corresponding P/N ratios. A positive result was defined as P/N ≥ 2.1. (**B**) SPR sensorgrams showing real-time interaction between KJJ4 and HTNV-Gn_19–371_.

We demonstrated that KJJ4 binds to inactivated whole-virus HTNV antigen, but whether it specifically targets Gn or Gc remained unclear. Previous reports indicate that amino acids 19–371 of Gn and 649–1105 of Gc are suitable regions for neutralizing activity studies [24,25,31]. Therefore, these regions were selected for expression and purification. Our results showed that KJJ4 bound weakly to Gc but exhibited dose-dependent binding to Gn. As shown in Figure 3A, KJJ4 bound to Gn_19–371_ with P/N ratios ≥ 2.1 at concentrations ≥ 32 ng/mL, indicating dose-dependent binding to Gn.

Surface plasmon resonance (SPR) was used to quantify the binding kinetic parameters between KJJ4 and Gn_19–371_. As shown in Figure 3B, the sensorgrams demonstrated specific, reversible binding: the response increased during the association phase and approached a plateau, then declined during the dissociation phase. The obtained kinetic parameters were: association rate constant = 4.70 × 10^3^ M^−1^s^−1^, dissociation rate constant = 3.06 × 10^−3^ s^−1^, and *K_D_* = 6.50 × 10^−7^ M (650 nM). The maximum binding response (Rmax) was approximately 14.9 RU, reflecting saturable binding under these conditions. Overall, the interaction exhibits modest affinity, with relatively slow association and measurable dissociation, consistent with a specific and reversible binding interaction.

## 3. Discussion

HFRS caused by HTNV remains a major public health burden in China, which accounts for more than 90% of global cases, with 576,361 cases reported between 1995 and 2020 [2,5,8,9,10]. Despite decades of clinical experience, management still relies largely on supportive care and off-label ribavirin, which has limited efficacy once disease progresses and is associated with significant adverse effects [11,32]. No FDA- or EMA-approved vaccines or antiviral therapeutics are currently available for HFRS, although HTNV/PUUV DNA vaccines have advanced to Phase 1–2a trials and induced neutralizing antibody responses in healthy volunteers [33,34]. In this therapeutic gap, passively administered neutralizing antibodies (NAbs) represent a rational strategy, particularly because HTNV RNA load during the early febrile/hypotensive phase correlates with disease severity and adverse outcomes [35]. Accordingly, reducing early viremia through glycoprotein-directed NAbs is biologically plausible and clinically relevant.

The present study identifies KJJ4 as a fully human anti-HTNV IgG4 antibody that binds monomeric Gn with modest affinity (*K_D_* = 650 nM) and neutralizes authentic HTNV in vitro (IC_50_ = 2.879 μg/mL). These findings extend a growing body of work showing that the hantavirus envelope glycoproteins—organized as tetrameric (Gn/Gc)_4_ spikes on the virion surface—are the principal targets of protective humoral immunity [24,36,37]. Recombinant HTNV Gn immunization elicits neutralizing responses in animal models and has enabled isolation of structurally defined antibodies such as HTN-Gn1 and nnHTN-Gn2, which map to distinct epitopes within the Gn head and helped establish Gn as a viable vaccine immunogen [24]. More recently, AH100, a human-origin anti-Gn mAb, was shown to neutralize HTNV with an IC_50_ of 1.07 ± 0.19 μg/mL and to bind Gn with higher apparent affinity (*K_D_* = 246.3 ± 55.4 nM) [25]. Crystallographic analysis revealed that AH100 recognizes domain A, the β-ribbon, and the E3-like domain of the Gn head and, when modeled on the (Gn/Gc)_4_ lattice, occupies a site between neighboring Gn protomers, where it likely blocks conformational changes required for entry [25]. Relative to AH100, KJJ4 displays weaker binding to monomeric Gn yet retains neutralizing activity in the low microgram-per-milliliter range, suggesting that absolute monomeric affinity alone is an incomplete predictor of antiviral potency.

An important interpretive issue is the apparent disconnect between SPR-measured binding and cell-based neutralization. SPR analysis reports equilibrium binding to soluble monomeric Gn_19–371_ (*K_D_* = 650 nM), whereas neutralization was measured against infectious HTNV on Vero E6 cells (IC_50_ = 2.879 μg/mL, corresponding to ~19 nM for a 150 kDa IgG). This ~30-fold difference is consistent with bivalent avidity on the native, multivalent Gn/Gc lattice of virions and therefore does not necessarily indicate weak functional engagement on intact particles. Similar principles have been emphasized for broadly neutralizing hantavirus antibodies: SNV-53 recognizes a quaternary epitope at the Gn/Gc interface, neutralizes principally through fusion inhibition, cross-protects against HTNV in vivo, and loses detectable neutralizing activity as a Fab fragment, indicating dependence on bivalent engagement [38]. ADI-42898, isolated from a PUUV-convalescent donor, binds a quaternary Gn/Gc epitope involving the Gn capping loop and Gc fusion loop, neutralizes diverse Old and New World hantaviruses, and protects rodents against ANDV and PUUV challenge [39,40]. SNV-42, a near-germline human mAb that binds the membrane-distal region of the Gn head and blocks both receptor recognition and membrane fusion, further illustrates that membrane-distal Gn epitopes can support potent neutralization [41]. Together, these studies indicate that in vivo protection depends not only on monomeric affinity but also on epitope geometry, spike context, and avidity. Whether the affinity and epitope specificity of KJJ4 are sufficient for therapeutic benefit therefore cannot be inferred from in vitro binding alone and must be tested in relevant animal models.

Because KJJ4 bound recombinant Gn_19–371_ but only weakly recognized Gc, its epitope most likely resides within the Gn head, the receptor-binding domain that is critical for viral attachment and entry [24,37]. In the current structural framework of hantavirus neutralization, vulnerable sites include: (i) outer Gn head epitopes such as those of HTN-Gn1 (loop β4–β5) and AH100 (domain A/β-ribbon/E3-like domain) [24,25]; (ii) quaternary Gn/Gc interface epitopes exemplified by ADI-42898 and SNV-53 [38,39,40]; and (iii) more cryptic Gc domain I/fusion-loop targets such as SNV-24 and HTNV antibody A5 [38,42]. AH100 occupies an inter-protomer site on the inner face of Gn, distinct from the outer HTN-Gn1 epitope [25], underscoring that multiple non-overlapping Gn-directed mechanisms can confer neutralization. Whether KJJ4 overlaps AH100/HTN-Gn1, engages the capping-loop region, or recognizes a separate Gn face remains unknown and should be addressed by competition ELISA/SPR with AH100 and HTN-Gn1, alanine scanning of surface-exposed Gn residues, and escape mutant selection. Defining the KJJ4 epitope is essential for comparing its mechanism with AH100 and for assessing resistance risk across natural HTNV isolates.

A notable feature of KJJ4 is its IgG4 format with the S108P hinge mutation, which was introduced to prevent Fab-arm exchange and stabilize the molecule [29,43]. IgG4 antibodies are generally considered poor activators of classical complement and can engage FcγRIIb, raising the possibility—still untested for KJJ4—of attenuating pathological immune activation while delivering antiviral neutralization [28,30,43]. This rationale is attractive in HFRS, which is increasingly understood as an immune-mediated disease in which viral replication and host inflammatory responses jointly determine severity [44,45,46]. Early HTNV RNA load correlates with disease severity and adverse outcome [35], and an ideal HTNV immunotherapeutic might therefore combine direct neutralization with mitigation of harmful host inflammation. However, because complement activation and FcγRIIb binding were not experimentally evaluated for KJJ4, any immunomodulatory advantage of the IgG4 scaffold remains speculative and should be tested directly, ideally in parallel with an IgG1 variant to separate Fc-dependent from Fc-independent effects.

When placed in the broader landscape of anti-hantavirus countermeasures developed since 2016, KJJ4 occupies an important but still early position. AH100 provides a human, HTNV-focused candidate with a defined Gn-head epitope and favorable in vitro potency [25]. ADI-42898 and SNV-53 exemplify the current benchmark for breadth and in vivo efficacy through quaternary spike recognition [38,39,40]. At the polyclonal level, SAB-163—a fully human, quadrivalent product targeting ANDV, SNV, HTNV, and PUUV—shows potent neutralization (PRNT_50_ > 200,000), favorable pharmacokinetics in hamsters (t½ ~10–15 days), and protection in HTNV, PUUV, SNV, and ANDV models when administered before or shortly after challenge; it has completed IND-enabling safety studies and represents the most advanced antibody-based medical countermeasure reported to date [47]. Compared with these candidates, KJJ4 offers a fully human monoclonal format and demonstrable in vitro HTNV neutralization, but lacks epitope definition, breadth assessment, pharmacokinetic profiling, and in vivo efficacy data. Its monomeric Gn affinity is lower than that of AH100, and cross-reactivity with other HFRS-associated hantaviruses such as PUUV and Dobrava virus remains untested.

Several limitations of the present work should be acknowledged. First, all functional conclusions are based on in vitro assays; therapeutic potential cannot be established without efficacy, pharmacokinetic, and toxicology studies in HTNV animal models [17,18,37,38]. Second, the epitope of KJJ4 has not been mapped, precluding mechanistic comparison with AH100, HTN-Gn1, ADI-42898, or SNV-53. Third, the immunological consequences of the IgG4-S108P scaffold were not examined. Fourth, potential cross-neutralization against heterologous hantaviruses was not assessed, limiting conclusions about geographic or strain coverage. Finally, although KJJ4 neutralizes HTNV at concentrations comparable to some reported human NAbs on a mass basis, head-to-head benchmarking against AH100, SAB-163, or broadly neutralizing mAbs under identical assay conditions has not been performed.

Future studies should prioritize epitope mapping and structure-guided mechanism analysis, followed by in vivo evaluation in Syrian hamster or other validated HTNV challenge models. Parallel testing of IgG4-S108P and IgG1 versions would clarify whether Fc effector functions contribute to protection or immunopathology in HFRS. Cross-reactivity panels against PUUV, Seoul virus, and Dobrava virus should be included to define the clinical niche of KJJ4, whether as a monotherapy, as part of a cocktail with complementary epitope specificity, or in combination with antivirals. Combining a Gn-head antibody such as KJJ4 with a Gn/Gc interface antibody may also improve therapeutic potency and resistance to escape. Addressing these questions will determine whether KJJ4 can progress from a promising in vitro reagent to a clinically meaningful component of HFRS preparedness. Ultimately, the translational value of KJJ4 will depend on whether its in vitro neutralization can be converted into viremia reduction and organ protection during the early febrile phase, when viral load is highest and intervention is most likely to alter disease course [35].

## 4. Materials and Methods

### 4.1. Cells and Virus

The Vero E6 cells used in this study were kindly provided by the Department of Microbiology, The Fourth Military Medical University (Xi’an, China). Vero E6 cells were routinely cultured in DMEM (high glucose, Gibco, Grand Island, NY, USA, Cat# 11965092) supplemented with 10% fetal bovine serum (FBS, Gibco, Grand Island, NY, USA, Cat# 10099141) and 1% penicillin–streptomycin (Gibco, Grand Island, NY, USA, Cat# 15140122), at 37 °C in a 5% CO_2_ humidified incubator. In our study, the cells were resuscitated, maintained, and processed by our co-author Yanbo Wang, who is responsible for all cell culture work described in the manuscript. Hantaan virus (HTNV) strain 76–118 was provided by the Department of Microbiology, Fourth Military Medical University, and viral titers were determined by plaque-forming assay.

### 4.2. Molecular Design and Gene Synthesis of KJJ3 and KJJ4 Antibodies

Using the human anti-HTNV Fab fragments 3–12 and 4–19, previously screened by phage display technology in our laboratory [27], as parental molecules, we performed directed molecular modification and full-gene synthesis. KJJ3 antibody: The two V_L_ domains of the Fab 3–12 were linked via a flexible (G_4_S)_3_ linker (GGGGS-GGGGS-GGGGS) to construct a V_L_-V_L_ tandem antibody, with a 10 × His tag fused to the C-terminus for protein purification and detection. KJJ4 antibody: Based on the gene sequence of Fab 4–19, the IgG4 subtype was selected as the constant region, and the S108P mutation was introduced to eliminate Fab-arm exchange. Light-chain and heavy-chain gene sequences of KJJ4 were designed separately. The full-length gene sequences of KJJ3 and KJJ4 were synthesized by Sino Biological Inc (Beijing, China).

### 4.3. Construction and Identification of Recombinant Expression Plasmids

Recombinant plasmids were constructed using restriction enzyme digestion and ligation. Specific primers containing appropriate restriction sites were designed based on the gene sequences of KJJ3, KJJ4 light chain, and KJJ4 heavy chain. Target gene fragments were amplified by PCR using the synthesized full-length genes as templates. The pPG3-L empty vector was digested with the same restriction enzymes, and the linearized vector was purified by gel extraction. The target fragment and linearized vector were mixed at a 1:3 molar ratio and ligated using T4 DNA ligase at 16 °C overnight to generate the recombinant plasmids pPG3-L-KJJ3, pPG3-L-KJJ4-LC, and pPG3-L-KJJ4-HC.

The ligation products were transformed into competent Escherichia coli DH5α (Takara, Kusatsu, Japan, Cat# 9057), plated on LB agar containing ampicillin, and cultured at 37 °C for 12 h. Single colonies were selected and expanded in LB liquid medium. Recombinant plasmids were extracted using a plasmid extraction kit (QIAprep Spin Miniprep Kit, QIAGEN, Hilden, Germany, Cat# 27104) and verified by PCR, double restriction enzyme digestion, and bidirectional Sanger sequencing (Sino Biological Inc, Beijing, China). Sequencing results were aligned with the designed gene sequences using DNAMAN 8.0 software (Lynnon Biosoft, San Ramon, CA, USA, RRID: SCR_014954) to confirm correct sequence, intact open reading frame, and absence of unintended mutations. Verified positive recombinant plasmids were then amplified in large quantities and stored at −20 °C for future use.

### 4.4. Eukaryotic Expression of Recombinant KJJ3 and KJJ4

HEK293 cells were cultured in DMEM supplemented with 10% FBS at 37 °C in a 5% CO_2_, >70% humidity atmosphere until reaching a density of approximately 5 × 10^5^ cells/mL. Cells in logarithmic growth phase with >95% viability were seeded at 2.0 × 10^6^ cells/mL in pre-warmed serum-free medium in 75 cm² tissue culture flasks with vented caps (Sangon Biotech, Shanghai, China, Cat# F605102-9001), with a working volume of 20–25% of the total vessel capacity. The culture was maintained in a constant-temperature shaker (Climo-Shaker ISF1-XC, Kuhner, Basel, Switzerland) at 37 °C, 5% CO_2_, >70% humidity, with shaking at 120–140 rpm for 18–24 h. When cell density reached 2.5–4.0 × 10^6^ cells/mL with ≥97% viability, transfection was performed. Plasmid DNA (1 μg per mL of final culture volume) and PEI transfection reagent (Polysciences, Warrington, PA, USA, Cat# 23966-2) were mixed at a 1:3 mass ratio. Both were diluted separately in equal volumes of serum-free medium, combined, mixed, and incubated at room temperature for 15 min to form complexes. The complexes were added dropwise to the cell culture. After 24 h, expression enhancer was added, and the culture was continued for 6 days. The supernatant was then collected by centrifugation at 2000× *g* for 20 min at 4 °C.

### 4.5. Affinity Purification and Validation of Recombinant KJJ3 and KJJ4

The collected supernatant was loaded at a constant flow rate onto an affinity column (GE Healthcare, Uppsala, Sweden, Cat# 17040201) pre-equilibrated with binding buffer at 4 °C. After loading, the column was washed with 5 column volumes of binding buffer to remove non-specifically bound proteins. The target antibody was eluted using elution buffer containing a competitive elution reagent, and peak fractions were collected. Purification efficiency was assessed by SDS-PAGE under both non-reducing and reducing conditions. For reducing conditions, samples were mixed with loading buffer containing dithiothreitol (DTT) and boiled to break interchain disulfide bonds; for non-reducing conditions, DTT was omitted. Gels were stained with Coomassie Brilliant Blue to visualize protein bands. Band intensities were quantified using ImageJ 1.54f (National Institutes of Health, Bethesda, MD, USA) to estimate purification purity. Purified antibody concentrations were determined using a BCA assay kit (Thermo Fisher Scientific, Waltham, MA, USA, Cat# 23225), and aliquots (1 mg per tube) were stored at −80 °C.

### 4.6. Binding Capacity to Inactivated HTNV Antigen

HTNV is effectively inactivated by autoclaving at 120 °C under high pressure for 30 min. Virus inactivation was performed using a method previously validated in our facility (inactivation was not re-verified in the present study). Complete inactivation was verified by blind serial passage of the autoclaved material in Vero E6 cells, with negative HTNV nucleocapsid immunostaining, because HTNV produces little or no cytopathic effect in this cell line and the absence of CPE alone is not a valid inactivation criterion. Inactivated HTNV antigen (TCID_50_ = 10^4^) was diluted 1:100 in coating buffer, added to 96-well plates at 200 μL/well, and incubated overnight at 4 °C. Plates were washed three times with PBST and blocked with 3% BSA in PBS (100 μL/well) at 37 °C for 1 h. After washing, KJJ3 and KJJ4 antibodies were serially diluted in PBS containing 0.1% BSA to eight concentrations (500,000, 100,000, 20,000, 4000, 800, 160, 32, 6.4 ng/mL), added at 100 μL/well, and incubated at 37 °C for 1 h. Negative control wells received PBS. After three PBST washes, HRP-conjugated secondary antibodies were added: for KJJ3, HRP-conjugated 6 × His monoclonal antibody (Proteintech, Wuhan, China, Cat# HRP-66005) diluted 1:5000; for KJJ4, HRP-conjugated goat anti-human IgG antibody (Proteintech, Wuhan, China, Cat# SA00001-17) diluted 1:1000. Plates were incubated at 37 °C for 40 min, washed five times with PBST, and incubated with 100 μL/well TMB substrate at 37 °C in the dark for 10 min. The reaction was stopped with 50 μL/well of 2 M H_2_SO_4_. After 5 min, OD_450_ was measured using a microplate reader (Synergy H1, BioTek Instruments, Winooski, VT, USA). A positive result was defined as a ratio of experimental well OD_450_ to negative control OD_450_ (P/N) ≥ 2.1.

### 4.7. Binding Capacity to Recombinant Gn_19–371_ Protein

Recombinant HTNV-Gn_19–371_ protein was diluted to 10 μg/mL in coating buffer, added at 100 μL/well to 96-well plates, and incubated overnight at 4 °C. Blocking, washing, and detection were performed as described in Section 4.6, with KJJ4 serially diluted to seven concentrations (100,000, 20,000, 4000, 800, 160, 32, 6.4 ng/mL). An IgG4 isotype control antibody was used as negative control.

### 4.8. In Vitro Microneutralization Assay

Vero E6 cells were seeded at 1.5 × 10^4^ cells/well in 96-well plates. KJJ3 antibody was serially diluted to seven concentrations (100, 20, 4, 0.8, 0.16, 0.032, and 0.0064 μg/mL); for KJJ4, eleven concentrations were tested (100, 33.3, 11.1, 3.7, 1.2, 0.41, 0.14, 0.046, 0.015, 0.005, and 0.0016 μg/mL), with a virus-only control included. Each antibody dilution was mixed with HTNV (MOI = 0.01) and incubated overnight at 4 °C. The antibody-virus mixtures were then added to the cells. After 4 h, the supernatant was discarded, and 2% carboxymethyl cellulose overlay medium (Sigma-Aldrich, St. Louis, MO, USA, Cat# C4888) was added. Plates were incubated at 37 °C for 5 days. After removing the overlay, plates were washed three times with PBS, fixed with 4% paraformaldehyde for 2 h at room temperature, permeabilized with 0.5% Triton X-100 for 12 min, and blocked with 3% BSA for 1 h. Primary antibody (anti-HTNV, 1:1000) was added and incubated overnight at 4 °C. After washing, HRP-labeled goat anti-mouse antibody (1:1000) was added for 4 h at room temperature. Plaques were visualized with TMB substrate and counted using an ELISPOT reader (AID iSpot Reader, Autoimmun Diagnostika, Strassberg, Germany). Percent neutralization was calculated relative to the virus-only control, and the half-maximal inhibitory concentration (IC_50_) was determined by nonlinear regression using GraphPad Prism 10.1.2 (GraphPad Software, Boston, MA, USA).

### 4.9. SPR Determination of Binding Kinetics

Binding kinetics between KJJ4 and HTNV-Gn_19–371_ were measured using a Biacore 8K SPR biosensor (Cytiva, Uppsala, Sweden, Cat# 29335949) at 25 °C. The running buffer was 1 × HBS-EP, and regeneration was performed with 10 mM glycine-HCl (pH 1.5). A Series S Protein A sensor chip (Cytiva, Cat# 29127555) was used for ligand capture. Serially diluted Gn_19–371_ (analyte) was flowed over flow cell 1 (blank control) and flow cell 2 (ligand-immobilized) at 30 μL/min. Association and dissociation phases were monitored, and sensorgrams were processed using Biacore Insight Evaluation Software 5.0.18 (Cytiva, Uppsala, Sweden). Double referencing (subtraction of flow cell 1 and blank injection signals) was applied. Kinetic parameters (ka, kd, *K_D_*) were obtained by fitting to a 1:1 binding model. Chi^2^ and Rmax were also analyzed.

### 4.10. Statistical Analysis

All experiments were independently repeated three times. Data are presented as mean ± SEM. Statistical analysis was performed using SPSS 26.0 (IBM, Armonk, NY, USA, RRID: SCR_002865). Group comparisons were conducted using one-way ANOVA followed by Tukey’s multiple comparison test. A *p* value < 0.05 was considered statistically significant.

## 5. Conclusions

In this study, we generated and experimentally characterized KJJ4, a fully human IgG4 (S108P) antibody targeting Hantaan virus glycoprotein Gn. KJJ4 bound recombinant Gn_19–371_ with a *K_D_* of 650 nM and neutralized HTNV infection of Vero E6 cells in vitro with an IC_50_ of 2.879 μg/mL, whereas the VL-VL tandem antibody KJJ3 showed only minimal neutralizing activity (IC_50_ = 27.12 μg/mL, approximately 9-fold weaker than KJJ4). The defining next steps are experimental: epitope mapping and mutagenesis of the Gn–KJJ4 interface, cross-reactivity testing against other pathogenic hantaviruses, and efficacy evaluation in HTNV animal models. These data position KJJ4 as a candidate for further preclinical development against HTNV-associated HFRS.

## Data Availability

The original contributions presented in the study are included in the article/Supplementary Materials. Further inquiries can be directed to the corresponding author.

## Supplementary Materials

The following supporting information can be downloaded at: https://www.mdpi.com/article/10.3390/ijms27187994/s1.
