# Supplementary material for: Generation and Characterization of the Human Anti-HTNV Antibody KJJ4
Source: Int J Mol Sci. 2026 Sep 8;27(18):7994. doi: 10.3390/ijms27187994 (PMC13606443; doi:10.3390/ijms27187994)
Supplement: Supplementary file 1 [file ijms-27-07994-s001.zip › ijms-4475144-supplementary.pdf]

Supplementary Materials:

Supplementary Table S1. Amino acid sequence of KJJ3 antibody.

Name	Antibody format	Amino acid sequence
KJJ3	V_L-V_L	DIQMTQSPDSLAVSLGERATINCRSSXXXXXXXXXXXXLAWYRQK<br>PGQPPKLLIYXXXTRESGVPDRISGSGSGTDFSLTISSLQAEDVAVY<br>YCXXXXXXXXXFGQGTKLEIKGGGGSGGGGSGGGGSDIQMTQSP<br>DSLAVSLGERATINCRSSXXXXXXXXXXXXLAWYRQKPGQPPKLLI<br>YXXXTRESGVPDRISGSGSGTDFSLTISSLQAEDVAVYYCXXXXXXX<br>XXFGQGTKLEIK

Note: The CDR regions are represented by “X” to protect the intellectual property of the related patent application.

Supplementary Table S2. Gene sequence of KJJ3 antibody.

Name	Antibody format	Amino acid sequence
KJJ3	V_L-V_L	GACATCCAGATGACCCAGTCTCCAGACTCCCTGGCTGTGTCTCTGG<br>GCGAGAGGGCCACCATCAACTGCAGGTCCAGC#TTAGCTTGGTATC<br>GCCAGAAACCAGGACAGCCTCCTAAGCTGCTCATTTAC#ACCCGGG<br>AATCCGGGGTCCCTGACCGAATCAGTGGCAGCGGGTCTGGGACAG<br>ATTTCTCTCTCACCATCAGCAGCCTGCAGGCTGAAGATGTGGCAGTT<br>TATTACTGT#TTCGGCCAAGGGACCAAGCTGGAGATCAAACGGCGG<br>CGGCGGCTCCGGCGGCGGCGGCTCCGGCGGCGGCGGCTCCGACAT<br>CCAGATGACCCAGTCTCCAGACTCCCTGGCTGTGTCTCTGGGCGAG<br>AGGGCCACCATCAACTGCAGGTCCAGC#TTAGCTTGGTATCGCCAG<br>AAACCAGGACAGCCTCCTAAGCTGCTCATTTAC#ACCCGGGAATCC<br>GGGGTCCCTGACCGAATCAGTGGCAGCGGGTCTGGGACAGATTTCT<br>CTCTCACCATCAGCAGCCTGCAGGCTGAAGATGTGGCAGTTTATTA<br>CTGT#TTCGGCCAAGGGACCAAGCTGGAGATCAAAC

Note: The CDR regions are represented by “#” to protect the intellectual property of the related patent application.

Supplementary Table S3. Amino acid sequences of KJJ4 antibody.

Name	Antibody format	Amino acid sequence	Name
KJJ4	IgG4<br>（S108P）	EIVLTQSPATLSLSPG<br>ERATLSCRASXXXXX<br>XLAWYQQKPGQAP<br>RLLIYXXXNRATGIP<br>ARFSGSGSGTDFTLT<br>ISSLEPEDFATYYCX<br>XXXXXXXXXFGQGT<br>KVDIKRTVAAPSVFI<br>FPPSDEQLKSGTASV<br>VCLLNNFYPREAKV<br>QWKVDNALQSGNS	QVQLQQWGAGLLKPSETLSLTCAVLXXXXX<br>XXXXYWSWIRQPPGKGLEWIGEXXXXXXXIY<br>NPSLKSRVTISVDTSKNQFSLRLSAVTAADT<br>AVYFCXXXXXXXXXXXXXXXXWGQGTM<br>VTVSSASTKGPSVFPLAPCSRSTSESTAALGC<br>LVKDYFPEPVTVSWNSGALTSGVHTFPAVL<br>QSSGLYSLSSVVTVPSSSLGTKTYTCNVDHKP<br>SNTKVDKRVESKYGPPCPPCPAPEFEGGPSV<br>FLFPPKPKDTLMISRTPEVTCVVVDVSQEDP<br>EVQFNWYVDGVEVHNAKTKPREEQFNSTY<br>RVVSVLTVLHQDWLNGKEYKCKVSNKGLP

		QESVTEQDSKDSTYS<br>LSSTLTLSKADYEKH<br>KVYACEVTHQGLSS<br>PVTKSFNRGEC	SSIEKTISKAKGQPREPQVYTLPPSQEEMTKN<br>QVSLTCLVKGFYPSDIAVEWESNGQPENNY<br>KTTPPVLDSDGSFFLYSRLTVDKSRWQEGNV<br>FSCSVMHEALHNHYTQKSLSLSLGK

Note: The CDR regions are represented by “X” to protect the intellectual property of the related patent application. The red “P” indicates the S108P mutation.

Supplementary Table S4. Gene sequences of KJJ4 antibody.

Name	Antibody format	Amino acid sequence	Name
KJJ4	IgG4<br>（S108P）	GAAATAGTACTT<br>ACACAGAGCCCC<br>GCAACACTCTCT<br>CTTTCCCCAGGC<br>GAGAGGGCAAC<br>ACTGTCCTGTAG<br>GGCCTCA#CTGG<br>CCTGGTACCAGC<br>AGAAGCCAGGG<br>CAGGCACCGAGG<br>TTGCTGATCTAC#<br>AATAGGGCAACC<br>GGCATTCCTGCT<br>CGCTTTTCTGGCT<br>CAGGCTCAGGTA<br>CAGACTTCACCC<br>TCACAATCAGCA<br>GTCTGGAGCCCG<br>AAGATTTCGCCA<br>CCTACTACTGC#T<br>TCGGCCAGGGCA<br>CCAAGGTGGATA<br>TCAAGCGTACGG<br>TGGCTGCACCAT<br>CTGTCTTCATCTT<br>CCCGCCATCTGA<br>TGAGCAGTTGAA<br>ATCTGGAACTGC<br>CTCTGTTGTGTGC<br>CTGCTGAATAAC<br>TTCTATCCCAGA<br>GAGGCCAAAGTA<br>CAGTGGAAGGTG<br>GATAACGCCCTC<br>CAATCGGGTAAC<br>TCCCAGGAGAGT<br>GTCACAGAGCAG<br>GACAGCAAGGA	CAGGTACAATTGCAACAATGGGGAGCGGG<br>ACTTCTCAAGCCATCCGAAACTCTCAGTCT<br>TACATGCGCCGTACTG#TACTGGTCATGGAT<br>AAGGCAACCCCCAGGTAAGGGATTGGAAT<br>GGATAGGCGAA#ATTTACAATCCTTCCCTG<br>AAGTCAAGGGTTACGATCTCCGTGGACACT<br>AGCAAGAATCAGTTCTCTCTGCGCCTCTCA<br>GCGGTGACAGCTGCCGACACAGCTGTATAT<br>TTCTGC#TGGGGGCAGGGTACCATGGTTACT<br>GTATCTAGCGCTAGCACCAAGGGCCCATCG<br>GTCTTCCCGCTGGCGCCCTGCTCCAGGAGC<br>ACCTCCGAGAGCACAGCCGCCCTGGGCTG<br>CCTGGTCAAGGACTACTTCCCCGAACCGGT<br>GACGGTGTCGTGGAACTCAGGCGCCCTGAC<br>CAGCGGCGTGCACACCTTCCCGGCTGTCCT<br>ACAGTCCTCAGGACTCTACTCCCTCAGCAG<br>CGTGGTGACCGTGCCCTCCAGCAGCTTGGG<br>CACGAAGACCTACACCTGCAACGTAGATC<br>ACAAGCCCAGCAACACCAAGGTGGACAAG<br>AGAGTTGAGTCCAAATATGGTCCCCCATGC<br>CCACCCTGCCCAGCACCTGAGTTCGAGGGG<br>GGACCATCAGTCTTCCTGTTCCCCCCAAAA<br>CCCAAGGACACTCTCATGATCTCCCGGACC<br>CCTGAGGTCACGTGCGTGGTGGTGGACGTG<br>AGCCAGGAAGACCCCGAGGTCCAGTTCAA<br>CTGGTACGTGGATGGCGTGGAGGTGCATAA<br>TGCCAAGACAAAGCCGCGGGAGGAGCAGT<br>TCAACAGCACGTACCGTGTGGTCAGCGTCC<br>TCACCGTCCTGCACCAGGACTGGCTGAACG<br>GCAAGGAGTACAAGTGCAAGGTCTCCAAC<br>AAAGGCCTCCCGTCCTCCATCGAGAAAACC<br>ATCTCCAAAGCCAAAGGGCAGCCCCGAGA<br>GCCACAGGTGTACACCCTGCCCCCATCCCA<br>GGAGGAGATGACCAAGAACCAGGTCAGCC<br>TGACCTGCCTGGTCAAAGGCTTCTACCCCA<br>GCGACATCGCCGTGGAGTGGGAAAGCAAT<br>GGGCAGCCGGAGAACAACTACAAGACCAC<br>GCCTCCCGTGCTGGACTCCGACGGCTCCTT

		CAGCACCTACAGCCTCAGCAGCACCCTGACGCTGAGCAAAGCAGACTACGAGAAACACAAAGTCTACGCCTGCGAAGTCACCCATCAGGGCCTGAGCTCGCCCGTCACAAAGAGCTTCAACAGGGGAGAGTGTTAG	CTTCCTCTACAGCAGGCTAACCGTGGACAAGAGCAGGTGGCAGGAGGGGAATGTCTTCTCATGCTCCGTGATGCATGAGGCTCTGCACAACCACTACACACAGAAGAGCCTCTCCCTGTCTCTGGGTAAATAA

Note: The CDR regions are represented by "#" to protect the intellectual property of the related patent application.

Author Contributions: Ziyan Chen and Yanbo Wang contributed equally to this work. They designed and performed the majority of experiments, including antibody expression and purification, ELISA, neutralization assays, SPR analysis, and bioinformatics analysis. They also analyzed the data and drafted the manuscript. Yongli Hou, Liang Fang, Linfeng Cheng, Yusi Zhang, and Chunmei Zhang assisted with cell culture, virus propagation, and the microneutralization assay. Yun Zhang and Ying Ma helped revise the manuscript. Kang Tang and Lihua Chen conceived and supervised the study, secured funding, provided resources, and finalized the manuscript. All authors reviewed and approved the final version.
